# Alteration of Homeostasis in Pre-osteoclasts Induced by *Aggregatibacter actinomycetemcomitans* CDT

**DOI:** 10.3389/fcimb.2016.00033

**Published:** 2016-03-31

**Authors:** Dione Kawamoto, Ellen S. Ando-Suguimoto, Bruno Bueno-Silva, Joseph M. DiRienzo, Marcia P. A. Mayer

**Affiliations:** ^1^Department of Microbiology, Institute of Biomedical Sciences, University of São PauloSão Paulo, Brazil; ^2^Department of Microbiology, School of Dental Medicine, University of PennsylvaniaPA, USA

**Keywords:** *Aggregatibacter actinomycetemcomitans*, cytolethal distending toxin, monocytes, osteoclast, RANKL

## Abstract

The dysbiotic microbiota associated with aggressive periodontitis includes *Aggregatibacter actinomycetemcomitans*, the only oral species known to produce a cytolethal distending toxin (AaCDT). Give that CDT alters the cytokine profile in monocytic cells, we aimed to test the hypothesis that CDT plays a role in bone homeostasis by affecting the differentiation of precursor cells into osteoclasts. Recombinant AaCDT was added to murine bone marrow monocytes (BMMC) in the presence or absence of RANKL and the cell viability and cytokine profile of osteoclast precursor cells were determined. Multinucleated TRAP^+^ cell numbers, and relative transcription of genes related to osteoclastogenesis were also evaluated. The addition of AaCDT did not lead to loss in cell viability but promoted an increase in the average number of TRAP^+^ cells with 1-2 nuclei in the absence or presence of RANKL (Tukey, *p* < 0.05). This increase was also observed for TRAP^+^ cells with ≥3nuclei, although this difference was not significant. Levels of TGF-β, TNF-α, and IL-6, in the supernatant fraction of cells, were higher when in AaCDT exposed cells, whereas levels of IL-1β and IL-10 were lower than controls under the same conditions. After interaction with AaCDT, transcription of the *rank* (encoding the receptor RANK)*, nfatc1* (transcription factor), and *ctpK* (encoding cathepsin K) genes was downregulated in pre-osteoclastic cells. The data indicated that despite the presence of RANKL and M-CSF, AaCDT may inhibit osteoclast differentiation by altering cytokine profiles and repressing transcription of genes involved in osteoclastogenesis. Therefore, the CDT may impair host defense mechanisms in periodontitis.

## Introduction

*Aggregatibacter actinomycetemcomitans* is considered to be the most prevalent member of the HACEK group formed by Gram-negative facultative anaerobic bacteria related to endocarditis (Paturel et al., 2004). This bacterium is also associated with aggressive periodontitis. The disease is characterized by rapid and severe bone resorption for the age of onset that may be mediated by bacteria and their products or by the activation of T cells that lead to an over-production of RANKL (receptor activator of nuclear factor-κβ ligand) and stimulation of local osteoclastogenesis (Teng et al., 2000; Kawai et al., 2006).

The production of cytolethal distending toxin (CDT), along with leukotoxin, is considered to be a primary virulence trait of strains of *A. actinomycetemcomitans* that are associated with the ability of the bacterium to evade host immune defenses (Henderson et al., 2010). The *A. actinomycetemcomitans* CDT is an AB_2_-type toxin (Shenker et al., 1999) that binds to lipid rafts in the plasma membrane of eukaryotic cells via the CDTC subunit (Boesze-Battaglia et al., 2009). The CDTB subunit is the active part of the *A. actinomycetemcomitans* toxin exhibiting DNA cleavage properties similar to those of DNase I (Lara-Tejero and Galán, 2000). AaCDT arrests eukaryotic cells at the G_0_/G_1_ or G_2_/M interphase of the cell cycle in most susceptible cell types. Studies of various types of cells indicate a range of sensitivities to the toxin. As in the case of other genotoxins, CDT-treated cells can under some conditions activate DNA damage repair responses and survive CDT intoxication (Frisan, 2016).

The AaCDT appears to be especially toxic for lymphocytes and epithelial cells (Ohguchi et al., 1998; Shenker et al., 2001; Belibasakis et al., 2004; Mise et al., 2005; Smith and Bayles, 2006) by activation of apoptosis mechanisms and blocking proliferation (Shenker et al., 2007). However, the data on susceptibility to CDT mediated apoptosis in non-proliferating monocytic cells are controversial (Rabin et al., 2009).

CDT intoxication can also result in impaired functions of the surviving target cell (Hickey et al., 2000; Ando-Suguimoto et al., 2014). Previous data revealed that AaCDT can target monocytic cells, such as macrophages, altering their phagocythic capacity and cytokines profiles (Akifusa et al., 2001; Mise et al., 2005; Fernandes et al., 2008; Ando-Suguimoto et al., 2014; Belibasakis and Bostanci, 2014). Osteoclast precursors derive from the monocyte /macrophage hematopoietic lineage and their differentiation into osteoclasts is mediated by the ligation of RANKL with its receptor RANK, which then activates different signaling pathways (Cicek et al., 2011).

Despite, the evidences of AaCDT is related to increased production of RANKL by fibroblasts (Belibasakis et al., 2005a,b), and *Haemophilus ducreyi* CDT in Jurkat T-cells (Belibasakis et al., 2008), the role of CDT in osteoclasts differentiation is still not elucidated. Cytokines play an important role in inflammatory bone destruction by upregulating the production of RANKL (Moon et al., 2013). *In vivo*, osteoclastogenesis is supported by T-cells, which produce not only RANKL, but cytokines, such as TNF-α (Brunetti et al., 2005) and IL-7 (Colucci et al., 2005), which are involved in the process. Moreover, osteoclast precursor cells express cytokine receptors as IL-1, TNF-α, IFN-γ, IL-6, and IL-17, which positively influence osteoclastogenesis (Boyle et al., 2003; Bishop et al., 2009; Belibasakis and Bostanci, 2014). On the other hand, osteoclastogenesis can be inhibited by IL-10, osteoprotegerin (OPG), transforming growth factor beta (TGF-β) and interferon regulatory factor 8 (IFR-8) (Boyle et al., 2003; Evans and Fox, 2007; Takayanagi, 2007; Zhao et al., 2009; Belibasakis and Bostanci, 2014).

Given the evidence, that the AaCDT may target monocytic cells and CDT intoxication results in an altered cytokine profile, we hypothesized that CDT plays a role in bone homeostasis by affecting the differentiation of precursor cells into osteoclasts. Thus, the aim of this study was to determine the effects of AaCDT intoxication on osteoclast precursors such as murine bone marrow cells (BMC).

## Materials and methods

### Recombinant cytolethal distending toxin from *A. actinomycetemcomitans*

His-tagged AaCDT subunits were purified from recombinant *E. coli* pET15b*cdtA, E. coli* pET15b*cdtB*, and *E. coli* pET15b*cdtC* cell lysates by affinity chromatography on NI-NDA columns (Life Technologies, Carlsbad, CA). Active AaCDT was prepared by combining CDTA-His_6_, CDTB-His_6_, and CDTC-His_6_ in a 1:1:1 molar ratio as described previously (Mao and DiRienzo, 2002; Cao et al., 2006; Ando-Suguimoto et al., 2014). The trimeric toxin was obtained after shaking the subunit suspension at 0°C for 1 h. Non-reconstituted subunits were removed using an Amicon centrifugal filter unit with a molecular weight cutoff of 50 kDa (Millipore, Bilerica, MA). Protein concentration was estimated using the Bradford assay (Bio-Rad Laboratories, Hercules, CA).

### Animals

Six- to eight-weeks-old male C57BL/6 mice were purchased from the Animal facility of isogenic mice, Immunology Department of Biomedical Sciences Institute, University of São Paulo (ICBUSP, São Paulo, SP, Brazil). Animals were maintained under specific pathogen-free conditions at the experimental facility, Department of Microbiology, ICBUSP, kept in a conventional room with a 12-h light–dark cycle at constant temperature, fed Nuvulab CR-1 (Quimtia, Colombo, PR, Brazil) and allowed water *ad libitum*. All experimental procedures were examined and approved by the Institutional Animal Experimentation Ethics Committee (Approval ID 162/09/CEEA).

### Bone marrow mononuclear cells (BMMC)

Bone marrow cells were obtained from the femur and tibiae of the C57BL/6 mice by flushing with Dulbecco's modified Eagle's medium [DMEM (Gibco, Grand Island, NY) supplemented with 10% fetal calf serum (FCS), sodium bicarbonate (2.2 g/ml) using an intradermal needle (BD precision glide). Cells were suspended in the same medium supplemented with penicillin (1664 U/ml) and streptomycin (745 U/ml).

BMMC were separated with Histopaque 1083 (Sigma—Aldrich, Saint Louis, CA) and re-suspended in α-MEM complete medium (Sigma—Aldrich) [supplemented with 15% fetal calf serum (FCS), sodium bicarbonate (2.2 g/ml), penicillin (100 U/ml), streptomycin (100 U/ml), and gentamicin (Sigma-Aldrich), L-Glutamin (200 mM) (Sigma-Aldrich), MEM non-essential amino acids 1% (Gibco)]. α-MEM complete medium was added with 20 ng/ml macrophage-colony-stimulating factor (M-CSF, (Peprotech, Rocky Hill, NJ) which promotes survival and proliferation of osteoclast precursors (Ross and Teitelbaum, 2005).

### Effect of AaCDT on BMMC

BMMC (2 × 10^5^) were cultured in 96-well plates (Corning—Costar) in α-MEM complete medium (Sigma—Aldrich) supplemented with 20 ng/ml M-CSF and with or without 50 ng/ml RANKL (Peprotech, Rocky Hill, NJ) (Axmann et al., 2009; Makihira et al., 2011). AaCDT was added to each well in concentrations of 0, 12.5, and 25 μg/ml. The cells were incubated at 37°C with 5% CO_2_ in a fully humidified atmosphere for 6 days and maintenance was done every 2 days by removing 50% of the culture medium and adding the same volume of medium and its supplements.

Negative control cells were cultured in medium containing RANKL (50 ng/ml) and 100 ng/ml osteoprotegerin (OPG, Peprotech, Rocky Hill, NJ), whereas positive control cells were cultured in medium containing an optimal concentration of RANKL (100 ng/ml). After 6 days of culture, cell viability, the number of TRAP—positive multinuclear cells and gene expression were determined.

### Cell viability assay (MTT ASSAY)

Cell viability was determined by the addition of 10% MTT solution (Sigma-Aldrich, 0.5 mg/ml in PBS). After a 3-h incubation period, the insoluble formazan crystals were dissolved in 10% SDS and optical density (OD) measured at 540 nm using a 680 micro-plate reader (BioRad, Hercules, CA). Data are shown as the mean ± SD of three wells for each exposure group.

### Trap-positive cell counting

The number of TRAP^+^ multinucleated cells was determined after fixation, stained using the TRAP staining kit (387A, Sigma—Aldrich) and examined by inverted light microscope (Nikon Eclipse TS100) using a × 40 objective. TRAP^+^ cells presenting 1–2, 3–4, ≥5 nuclei were counted in each well, and the mean and standard deviation of the number of TRAP^+^ multinucleated cells per well calculated for each condition, in triplicate assays.

### Cytokine production

The production of IL1-β, TNF-α, IL-10 IL-6, and TGF-β was determined in cell supernatants using enzyme-linked immunosorbent assay (ELISA) commercial kits (Peprotech, Rocky Hill, NJ and R&D Systems, Minneapolis, MN—for TGF-β). Absorbance values were read at 405 nm against a standard curve and data expressed in ng/ml.

### Relative expression of genes associated with osteoclastogenesis

The cells were washed with PBS and total RNA was extracted using Trizol reagent (Ambion/Life Technologies). RNA yield and purity were determined using a spectrophotometer (Nanodrop ND 1000, Peqlab, Erlangen, Germany). Purified RNA served as template for the synthesis of cDNA using Superscript III First Strand Synthesis System for RT-PCR (Invitrogen).

Transcription levels were determined by qPCR using cDNA as a template in TaqMan inventoried assays for the *rank* (Mm 00437135_m1), *ctpk* (Mm 00484039_m1), *nfatc-1* (Mm 00479445 _m1), *irf-8* (Mm 00492567_m1), and *gapdh* (Mm 99999915_g1) genes (Applied Biosystems). TaqMan Fast Universal PCR Master Mix was added to the reactions (Applied Biosystems – Foster, CA). PCR reactions were performed using an initial incubation at 50°C for 2 min followed by 50 cycles of 95°C for 10 min, 95°C for 15 min, and 60°C for 1 min, in a thermocycler (Step One Plus Real-Time PCR system, Applied Biosystems).

The analysis of the relative quantification of genes related to osteoclastogenesis was performed by using the ΔΔCt Method (Pfaffl, 2001). Relative gene expression was determined after normalization of data to transcription levels of the housekeeping gene *gapdh* (glyceraldehyde-3-phosphate dehydrogenase) in each sample.

### Statistical analysis

Differences in cell viability and production of cytokines among groups were determined using ANOVA followed by Tukey. Values were considered significant when *p* < 0.05. For gene transcription profiles, Two-way ANOVA followed by Bonferroni was used to assess differences between control and experimental groups using mean CT-values derived from the triplicate samples. Differences in gene expression were considered to be significant when *p* < 0.05. For all analysis, Graphpad Prism version 4.0 was used (La Jolla, CA).

## Results

### Cell viability assay (MTT ASSAY)

**Figure 1:**
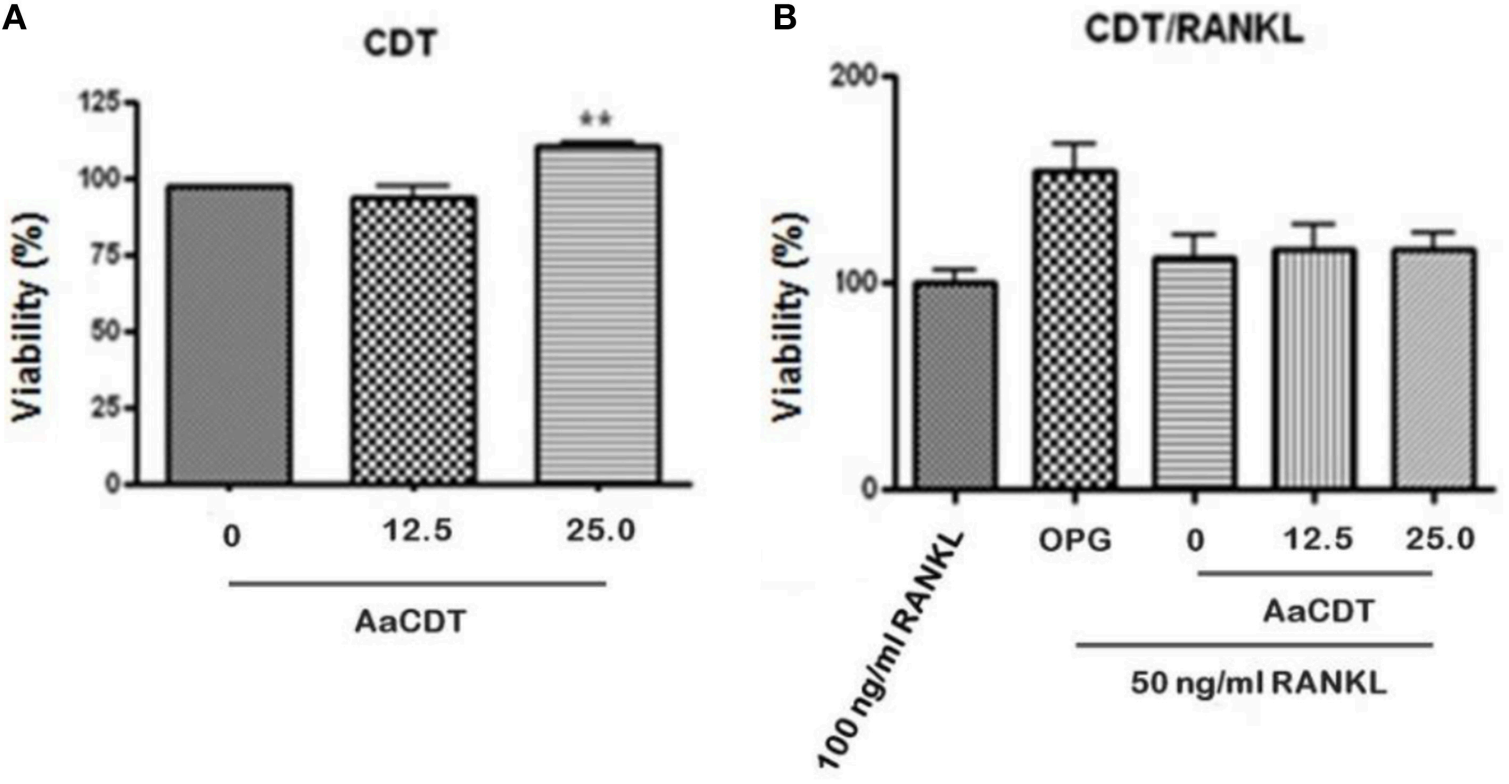
**Effect of recombinant AaCDT on monocyte viability after 6 days of incubation. (A)** without RANKL; **(B)** 50 ng/ml of RANKL. Cultures received either 100 ng/ml RANKL; 100 ng/ml OPG, or 50 ng/ml of RANKL. Cultures containing the lower concentration of RANKL also received either 0, 12.5, or 25.0 μg/ml of AaCDT. Statistically significant difference when compared to the culture lacking CDT (ANOVA-Tukey), ^**^p < 0.01.

Treatment of monocytes with the AaCDT (12.5 and 25.0 μg/ml) for 6 days did not result in a loss of viability when RANKL was omitted from or included in cultures (see Figures 1A,B, respectively). Addition of 25.0 μg/ml of AaCDT resulted in a slight but significant increase in BMMC viability when RANKL was not added.

### Trap-positive cell counting

**Figure 2:**
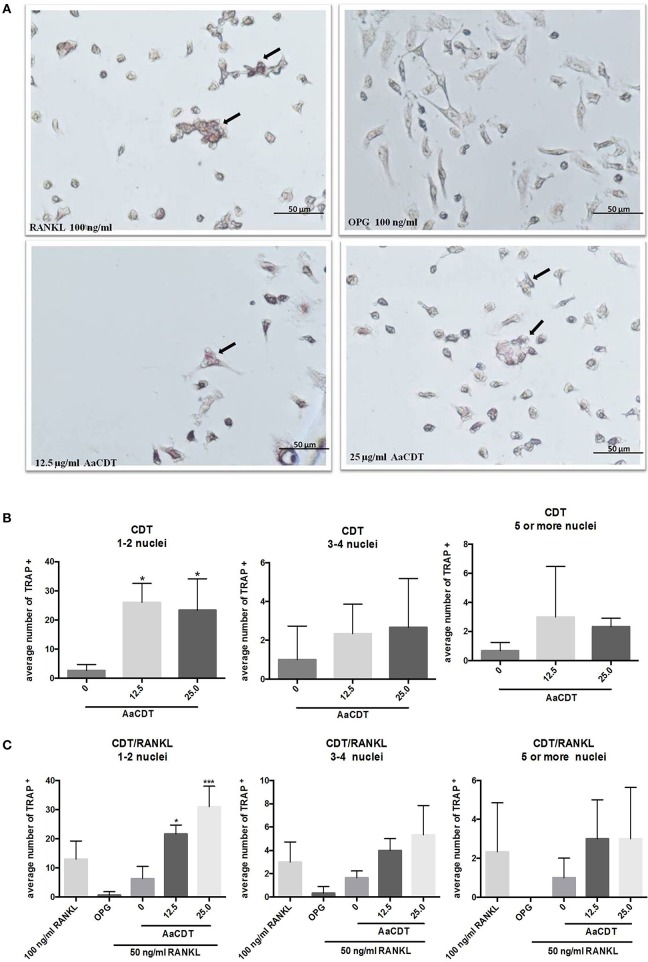
**Effect of AaCDT on the number of TRAP^+^ multinucleated cells**. In **(A)** a representative photomicrograph illustrating TRAP stained BMMC cells after 6 days of incubation with 50 μg/ml RANKL, and different concentrations of AaCDT. Image magnification is 40x using Olympus BX60 microscope and NIS-Elements F capture system (Nikon, Center Valley, PA, USA). Cells added with osteoprotegerin (OPG; 100 ng/ml) were used as negative controls or RANKL (100 ng/ml) used as positive control. In **(B)** without, in **(C)** with the addition of 50 ng/ml of RANKL the average of TRAP^+^ cells per well. Controls (without AaCDT): (0) negative control; (OPG) cells added with OPG 100 ng/ml; (100 ng/ml RANKL) cells added with optimal RANKL concentration for osteoclastogenesis. Statistically significant difference when compared with negative control (ANOVA-Tukey), ^*^p < 0.05, ^**^p < 0.01 and ^***^p < 0.001.

The addition of 12.5 and 25.0 μg/ml AaCDT led to an increase in the number TRAP^+^ cells harboring one or two nuclei in cultures lacking RANKL (Figure 2B), and in presence of RANKL the AaCDT. The exposure to increasing concentrations of AaCDT resulted in increased numbers of TRAP^+^ cells containing 3–4 nuclei, in a dose dependent way (Figure 2C), although these differences were not statistically significant. TRAP^+^ cells containing ≥3 nuclei as shown in Figure 2A, indicated that pre-osteoclasts differentiated to osteoclasts. The exposure to AaCDT in the presence of RANKL resulted also in increased numbers of TRAP^+^ cells containing ≥5 nuclei (not significant).

### Cytokine production

**Figure 3:**
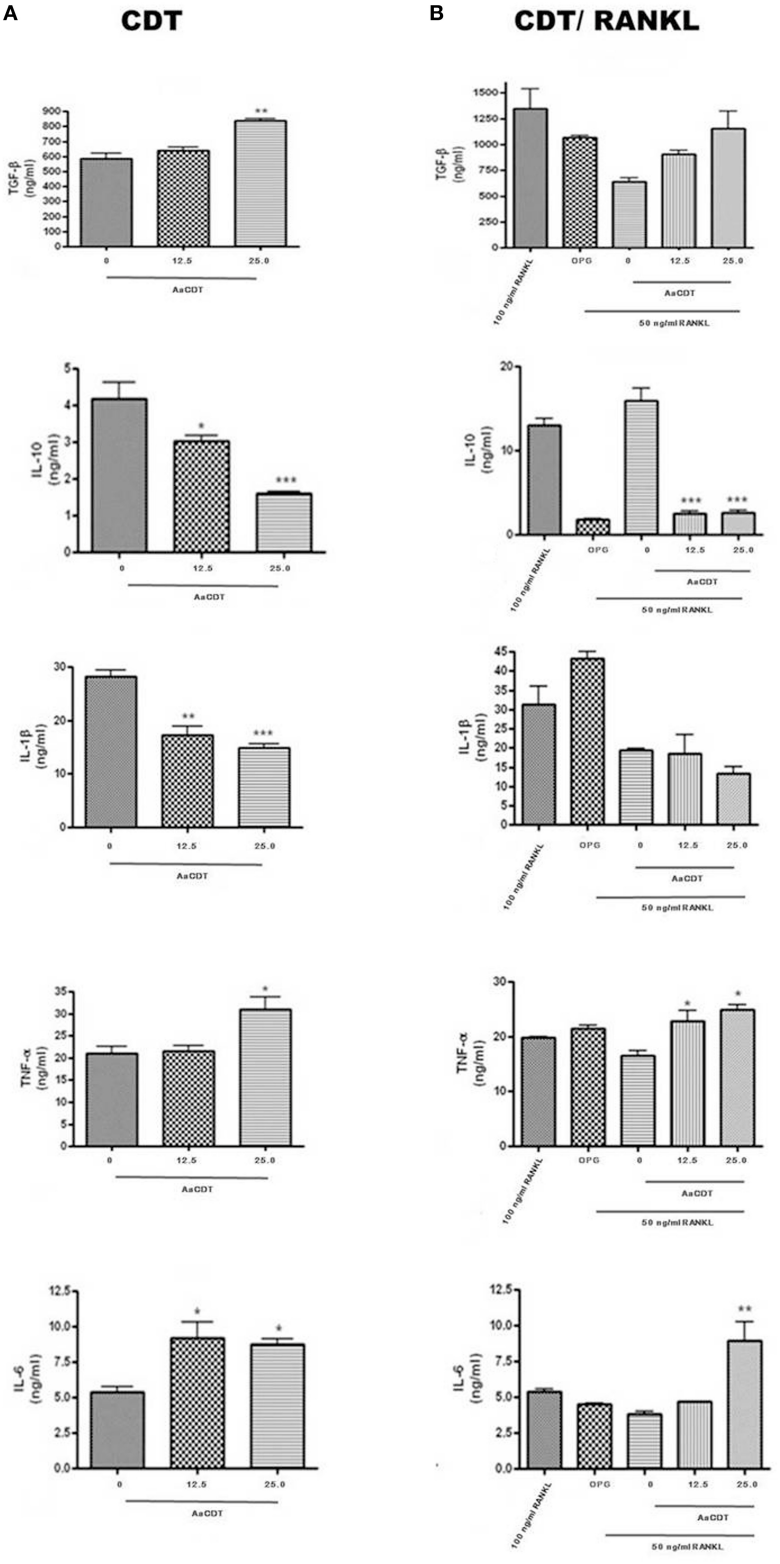
**Cytokines levels (TGF-β, IL-1β, TNF-α, and IL-6) in cell supernatant of BMMC added with AaCDT (12.5 and 25.0 μg/ml) after 6 days of incubation, in the absence (A) and presence (B) of 50 ng/ml RANKL**. Controls consisted of cells without AaCDT): (0) negative control; (OPG) cells added with 100 ng/ml osteoprotegerin; (100 ng/ml RANKL) cells added with optimal RANKL concentration for osteoclastogenesis. Statistically significant difference (ANOVA-Tukey) when compared with negative control: ^*^p < 0.05, ^**^p < 0.01 and ^***^p < 0.001.

After 6 days of incubation of monocyte with 12.5 or 25.0 μg/ml of AaCDT in absence of RANKL (Figure 3A) and addition of RANKL (Figure 3B), resulted in increased production of TGF-β and decreased production of IL-1β in the cultures without RANKL, but in the presence of RANKL, AaCDT exerted no effect on cytokines levels.

Furthermore, exposure to AaCDT resulted in increased levels of TNF-α and IL-6, and decreased levels of IL-10 in both assays, with and without RANKL. Data are shown from one assay in triplicate. The most dramatic changes promoted by AaCDT was observed for IL-6, which levels doubled in cells receiving 12.5 and 25.0 μg/ml AaCDT with and without the addition of RANKL, respectively. Furthermore, the levels of IL-10 were decreased by 50% in cells without RANKL exposed to 25.0 μg/ml of AaCDT, and by 90% in cells with RANKL exposed to 12.5 and 25.0 μg/ml of AaCDT.

### Relative expression of genes associated with osteoclastogenesis

BMMC were exposed to AaCDT at 12.5 or 25.0 μg/ml for 2, 4, and 6 days, in the presence or absence of RANKL, and gene expression was determined. AaCDT did not significantly alter expression of genes associated with osteoclastogenesis in the absence of RANKL (Data not shown).

Relative transcription of genes associated with osteoclastogenesis, including the transcription factor *nfatc-1* and genes encoding the receptor RANK and the protease cathepsin K, characteristic of osteoclasts, were increased in BMMC treated with RANKL. Transcription levels of these genes were more pronounced in the cells incubated with the optimal concentration of RANKL (100 ng/ml) after 2 and 4 days, whereas the addition of the suboptimal RANKL concentration (50 ng/ml) resulted in a delay in the transcription of genes associated with osteoclastogenesis. Transcription of the regulatory factor *ifr-8*, which interrupts osteoclastogenesis, was induced in cells submitted to 100 and 50 ng/ml RANKL after the transcription of genes associated with osteoclastogenesis. The relative expression of *ifr-8* was also high for the RANKL treated- cells submitted to OPG in all studied periods.

**Figure 4:**
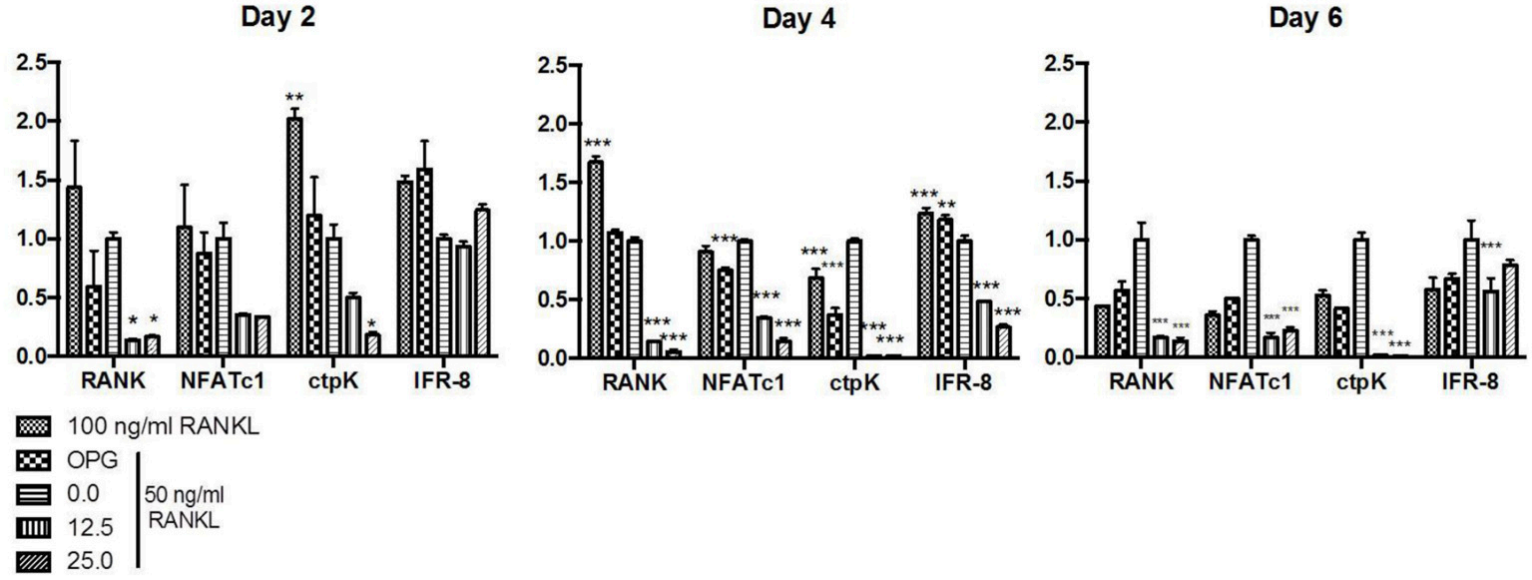
**Relative expression genes associated with osteoclastogenesis (*rank*, *nfatC1*, *ctpK*, and *ifr-8*) in monocytes submitted to RANKL (50 ng/ml) and AaCDT (12.5 and 25.0 μg/ml), after 2, 4, and 6 days: Controls (without AaCDT): (0) negative control; (OPG) cells added with 100 ng/ml OPG; (100 ng/ml RANKL) cells added with optimal RANKL concentration for osteoclastogenesis**. Statistically significant difference when compared with negative control ^*^p < 0.05; ^**^p < 0.01 and ^***^p < 0.001 (Two way ANOVA—Bonferroni).

The transcription of all studied genes associated with osteoclastogenesis in RANKL treated cells was always lower for those cells exposed to CDT than to control cells treated only with the same amount of RANKL (50 ng/ml) and even lower than for those exposed to the RANKL and the osteoclastogenesis inhibitor osteoprotegerin. Transcription analysis of cells treated with RANKL and exposed to AaCDT for two days revealed a downregulation of *rank* and *cptK* when compared with cells not exposed to AaCDT, and this downregulation persisted till the end of the experimental period (6 days). Furthermore, the transcription of the regulatory gene *ifr-8* was always low for the AaCDT treated cells (Figure 4).

## Discussion

The cytolethal distending toxin has a wide distribution among pathogenic Gram-negative bacteria (Guerra et al., 2011) and is frequently associated with virulence (Fabris et al., 2002; Höglund Åberg et al., 2013; Baig et al., 2015; Oloomi et al., 2015). The *cdt* operon is located in regions of the *A. actinomycetemcomitans* genome that contain elements suggestive of horizontal gene transfer (Mayer et al., 1999; Kittichotirat et al., 2016) and its maintenance in most strains of this species may be indicative of an ecological advantage.

In the present study the influence of recombinant AaCDT on osteoclast differentiation, as promoted by RANKL, was determined and resulted in no loss of viability. This result was consistent with the finding of other studies showing that non-proliferating cells are relatively resistant to CDT-induced apoptosis (Shenker et al., 2014).

The detection of TRAP^+^ cells with 1–2 nuclei and more than three nuclei when monocyte cultures were exposed to AaCDT (25.0 μg/ml), specially in the present of RANKL suggests that AaCDT potentiates the osteoclastogenic effect of RANKL. *In vitro* studies have indicated that the first TRAP^+^ multinucleated cells containing three to four nuclei appear between the 2nd and 4th day of culture with RANKL, while giant multinucleated cells, producing markers of mature osteoclasts such as cathepsin K, were only detected on day 5 (Makihira et al., 2011). Data on osteoclast precursor cells revealed that only cells with more than 10 nuclei were able to reabsorb bone (Lees et al., 2001). Thus, our data indicated that the effect of AaCDT on osteoclast differentiation was more pronounced in the initiation of the process, but AaCDT did not lead to significant increase in TRAP^+^ cells with ≥5 nuclei, indicating that it may not lead to differentiation into mature osteoclasts.

Osteoclast differentiation is a complex activity that is not only dependent on an interaction with RANKL (Wythe et al., 2014) but also that pro-inflammatory cytokines influences the process (Takayanagi, 2010). The CDT-intoxicated osteoclast precursors produced increased levels of TGF-β whereas levels of IL-1β decreased indicating that these cells produced an altered cytokine repertoire characteristic of osteoclastogenesis inhibition. TGF-β is able to inhibit osteoclast formation and decrease the lifetime of mature osteoclasts (Weitzmann et al., 2000) whereas IL-1β synergizes with RANKL to promote later stages of osteoclast differentiation (Lee et al., 2010).

On the other hand, AaCDT also led to increased levels of TNF-α as previously shown for the monocytic cell lineage THP-1 (Ando-Suguimoto et al., 2014). TNF-α prolongs osteoclasts survival (Lee et al., 2001) and induces the transcription factor NFATc-1 (Kim et al., 2008) which, in turn, induces osteoclastogenesis. Levels of IL-6 also increased after CDT intoxication in pre-osteoclastic cells. The effect of IL-6 on osteoclast precursor differentiation is dual since IL-6 can exhibit both inhibitory and stimulatory effects (Yoshitake et al., 2008; Axmann et al., 2009).

Our data indicated that IL-10 levels were decreased with high doses of AaCDT after 6 days of incubation. These data may seem contradictory since IL-10 is known to inhibit osteoclastogenesis (Wei et al., 2005; Mohamed et al., 2007; Takayanagi, 2010; Ivashkiv et al., 2011). However, it should be pointed out that CDT induces IL-10 production in monocytic cells after 2 days of intoxication (Akifusa et al., 2001), as expected, due to its phosphatase activity, similar to SHIP (Hazeki et al., 2007, 2011). Thus, a feedback effect may possibly result in the low IL-10 levels observed in CDT-intoxicated cells at the later stages of incubation.

In order to demonstrate if mature osteoclasts were formed from CDT-intoxicated pre-osteoclasts having an altered cytokine profile, the transcription of genes associated with osteoclastogenesis were also evaluated. The addition of recombinant AaCDT to RANKL treated monocytes led to down regulation of transcription of the *rank* and *ctpk* genes after 2 days of exposure (Figure 4), and this down regulation persisted till the end of the experiment (day 6), indicating that AaCDT repressed the transcription of these genes at the beginning of the osteoclastogenesis process.

Osteoclastogenesis requires the connection of M-CSF with its M-CSFR receptor, which activates proliferation and survival of osteoclast precursors (Tanaka et al., 2006). Then, a cascade in which RANK-RANKL (Receptor activation of NF-κB and its ligand) signaling pathway leads to activation of NF-κB, followed by the activation of molecular adaptor Grb-2-associated binder two (GAB2), which induces the activation of the transcription factor NFATc-1, mitogen-activated protein kinases (MAPKs), C-SRC, and AKT (Boyle et al., 2003). These events promote the osteoclast precursors to differentiate into mononuclear osteoclasts. NFATc-1 then induces the formation of multinuclear mature osteoclasts, characterized by the cathepsin K production (Matsumoto et al., 2004), carbonic anhydrase II and H ± ATPase (Asagiri and Takayanagi, 2007), DC-STAMP, and TRAP (Zhang et al., 2014) at the binding site with mineralized tissue (Teitelbaum, 2000), resulting in bone resorption. NFATc-1 is negatively regulated by IFR-8 (Hu et al., 2010). Furthermore, osteoclastogenesis can be inhibited by osteoprotegerin which binds to RANKL, preventing its ligation with RANK in the cell membrane (Lacey et al., 1998).

After RANK-RANKL interaction, NFATc-1 is expressed in the cytoplasm within 24 h and its nuclear translocation is more evident after 48 h, when cell fusion characteristic of osteoclasts starts, and multi-nucleated TRAP^+^ cells reach the maximum expression of TRAP and NFATc-1 after 72 h (Takayanagi et al., 2002).

Thus, our data evidences that the exposure of osteoclast precursors to M-CSF and RANKL concomitantly to CDT intoxication resulted in initial osteoclastogenesis events, as shown for increased number of TRAP^+^ cells, especially those with 1–2 nuclei. However, the CDT intoxicated cells were unable to continue differentiating into osteoclasts, associated with an environmental shift promoted by the release of inhibitory cytokines and repression of RANK expression. These events were followed by the inhibition of the transcription factor *nfatc-1*, and subsequent inhibition of genes encoding factors characteristic of mature osteoclasts, such as *cathepsin K*.

These observations are in accordance with recent data showing that CDT causes perturbation of phosphatidylinositol-3-kinase (PI-3K)/AKT signaling (Shenker et al., 2016), and it is known that AKT plays a role in osteoclast survival (Lee et al., 2001) and differentiation (Boyle et al., 2003).

These data may conflict with the bone destruction seen in aggressive periodontitis. However, imbalanced osteoclastogenesis is a process resultant from an exacerbated inflammatory process, with deleterious effects to the infectious agent. Thus, the inhibition of osteoclastogenesis promoted by AaCDT may represent its modulatory effect on host immune defenses.

## Author contribution

In this study, DK designed and performed experiments, analyzed data, and wrote the manuscript, ES and BS helped in experiments and did a critical revision. ES also helped in data analysis. JD did the critical revision and edits the manuscript. MM Supervised development of work, study design, helped in data interpretation and manuscript evaluation, and did the critical revision.

## Funding

This work was financed by São Paulo Research Foundation (FAPESP) Grant 09/54849-1. DK was supported by Coordenação de Aperfeiçoamento de Pessoal de Nível Superior (CAPES).

### Conflict of interest statement

The authors declare that the research was conducted in the absence of any commercial or financial relationships that could be construed as a potential conflict of interest.
